# Increased Arginase Expression and Decreased Nitric Oxide in Pig Donor Lungs after Normothermic Ex Vivo Lung Perfusion

**DOI:** 10.3390/biom10020300

**Published:** 2020-02-14

**Authors:** Farshad Tavasoli, Mingyao Liu, Tiago Machuca, Riccardo Bonato, David R. Grant, Marcelo Cypel, Shaf Keshavjee, Hartmut Grasemann

**Affiliations:** 1 Translational Medicine, SickKids Research Institute, The Hospital for Sick Children, Toronto, ON M5G 0A4, Canada; f_tavasoli@yahoo.ca; 2 Institute of Medical Science, University of Toronto, Toronto, ON M5S 1A8, Canada; Mingyao.Liu@utoronto.ca (M.L.); Marcelo.Cypel@uhn.ca (M.C.); Shaf.Keshavjee@uhn.ca (S.K.); 3 Latner Thoracic Surgery Research Laboratories, Toronto General Research Institute, Toronto, ON M5G 2C4, Canada; Tiago.machuca@surgery.ufl.edu (T.M.); riccardo.bonato@uhn.ca (R.B.); 4 Toronto Lung Transplant Program, University Health Network, Toronto, ON M5G 2C4, Canada; 5 Department of Surgery, Toronto General Hospital, Toronto, ON M5G 2C4, Canada; david.grant@uhn.ca; 6 Division of Respiratory Medicine, Department of Paediatrics, The Hospital for Sick Children, Toronto, ON M5G 1X8, Canada

**Keywords:** lung transplantation, ex vivo lung perfusion, nitric oxide, arginase

## Abstract

An established pig lung transplantation model was used to study the effects of cold ischemia time, normothermic acellular ex vivo lung perfusion (EVLP) and reperfusion after lung transplantation on l-arginine/NO metabolism in lung tissue. Lung tissue homogenates were analyzed for NO metabolite (NOx) concentrations by chemiluminescent NO-analyzer technique, and l-arginine, l-ornithine, l-citrulline and asymmetric dimethylarginine (ADMA) quantified using liquid chromatography-mass spectrometry (LC-MS/MS). The expression of arginase and nitric oxide synthase (NOS) isoforms in lung was measured by real-time polymerase chain reaction. EVLP preservation resulted in a significant decrease in concentrations of NOx and l-citrulline, both products of NOS, at the end of EVLP and after reperfusion following transplantation, compared to control, respectively. The ratio of l-ornithine over l-citrulline, a marker of the balance between l-arginine metabolizing enzymes, was increased in the EVLP group prior to reperfusion. The expression of both arginase isoforms was increased from baseline 1 h post reperfusion in EVLP but not in the no-EVLP group. These data suggest that EVLP results in a shift of the l-arginine balance towards arginase, leading to NO deficiency in the lung. The arginase/NOS balance may, therefore, represent a therapeutic target to improve lung quality during EVLP and, subsequently, transplant outcomes.

## 1. Introduction

Endogenous nitric oxide (NO) is important in the regulation of various physiological and patho-physiological conditions, including airway smooth muscle tone, vascular resistance and immune responses [1,2,3]. NO is produced by nitric oxide synthases (NOSs) that catalyze the reaction of l-arginine to l-citrulline. The constitutive NOS isoenzymes, neuronal NOS (nNOS) and endothelial NOS (eNOS) can be distinguished from inducible NOS (iNOS). The expression of iNOS is increased in response to inflammatory stimuli [2,3,4]. NOS activity depends on the presence of substrate l-arginine, and low l-arginine availability can cause NOS uncoupling and consequent formation of reactive oxygen species and peroxynitrite [4,5]. Limitation of l-arginine availability by arginase is an important post-transcriptional regulatory mechanism for NOS activity in different cardio-vascular and pulmonary conditions [6,7,8,9,10]. Arginase, which exists in two isoforms, also uses l-arginine as substrate to produce l-ornithine and urea [3,4].

Previous studies have suggested that alterations in the l-arginine/NO metabolism may contribute to complications after lung transplantation [11]. For instance, NO released by macrophages and neutrophils may contribute to the pathogenesis of ischemia reperfusion (I/R) injury [12], and the reaction of NO with oxygen species results in the formation of toxic radicals, which are critical in the development of I/R injury and primary graft dysfunction (PGD) [13,14]. However, some studies have suggested reduced NOS activity and NO deficiency following lung transplantation [15]. Inhaled NO improves gas exchange and oxygenation properties, i.e., the ratio of arterial oxygen tension to inhaled oxygen fraction (PaO_2_/FiO_2_) after lung transplantation [16], and can be used as a therapeutic intervention to successfully manage PGD in selected patients [17,18,19]. In a rabbit model of lung transplantation, administration of the NO-precursor l-arginine during reperfusion reduced I/R injury after cold ischemia by preventing vascular endothelial dysfunction [20]. Similarly, l-arginine supplementation during warm reperfusion following 12 h of cold ischemia time in neonatal piglet lungs resulted in improved pulmonary function as measured by partial oxygen pressure and lung compliance [21].

Normothermic ex vivo lung perfusion (EVLP) renders physiological conditions to maintain normal cellular metabolism in the donor organ and thereby provides the opportunity to evaluate lung function, tissue recovery and therapeutic interventions prior to transplantation, without increasing the risk of post-transplant complications [22,23,24,25,26,27,28,29]. EVLP in human lung transplantation is associated with non-inferior outcomes compared to standard organ preservation [27], and inhaled NO during EVLP was shown to improve oxygenation and pulmonary artery blood flow after transplantation of lungs from non-heart-beating donors in the rat [30].

The underlying reasons for decreased NO production after lung transplantation and the effect of EVLP on pulmonary NO formation are unknown but may include modifiable changes in the l-arginine/NO metabolism. We, therefore, studied the pulmonary l-arginine/NO metabolism in the immediate pre- and post-transplantation period utilizing an established pig model of normothermic EVLP and lung transplantation.

## 2. Materials and Methods

### 2.1. Animals

Domestic male Yorkshire pigs (25 to 35 kg) were treated in compliance with the “Principles of Laboratory Animal Care” prepared by the National Society for Medical Research and the “Guide for the Care of Laboratory Animals” by the National Institutes of Health. The experimental protocols were approved by the Animal Care Committee of the Toronto General Hospital Research Institute. The animal procedures were performed at the Latner Thoracic Surgery Research Laboratories, University Health Network—MaRS Centre, Toronto Medical Discovery Tower, Toronto General Hospital, Toronto, Canada.

Pig lungs were harvested and transplanted as previously described [31]. Briefly, explanted left lungs were kept at 4 °C (cold ischemia time, CIT) for 6 h prior to 12 h of normothermic acellular ex vivo lung perfusion (EVLP), followed by transplantation (EVLP group). For comparison, a second group of donor left lungs were transplanted after 18 h of cold preservation and no-EVLP (18 h CIT) [25,32,33]. Lung tissue samples were taken from Perfadex^®^-flushed lungs immediately after retrieval (0 h CIT), 18 h CIT (18 h CIT), and after EVLP (6 h CIT + 12 h EVLP). After transplantation, additional samples were taken 1 h after reperfusion in both the no-EVLP (18 h CIT/1 h post rep) and the EVLP (EVLP/1 h post rep) groups, as well as from recipient left lungs immediately after removal (normal control). All samples were frozen and kept at −80 °C prior to further processing.

### 2.2. Tissue Processing and Analyses

Lysis buffer, containing 25 mM Tris-HCl (pH = 7.4), 10% glycerol and 1% Triton X100, 1 mM phenylmethylsulfonyl fluoride (PMSF) (Calbochem, LaJolla, CA, USA), 2 mM ethylenediaminetetraacetate (EDTA), 2 μg/mL Pepstatin A, 2 μg/mL Leupeptin and 1 mM Dithiothreitol (DTT), was added to lung tissue samples in a weight-to-volume ratio of approximately 1:5. Samples were chopped and homogenised with a handheld rotor-stator (Polytron PT 1200E, Kinematica AG, Switzerland). After keeping homogenates on ice for one hour, samples were centrifuged for 20 min at 14,500 RPM and 4 °C. The supernatant was stored at −80 °C. Bradford protein assay was used for measurement of the protein content [34].

Total nitric oxide metabolite (nitrate+nitrite, NOx) concentrations were measured in lung tissue homogenates using chemiluminescent NO-analyzer techniques (NOA280, Eco Physics, Durnten, Switzerland) as previously reported [35]. l-arginine, l-ornithine, l-citrulline and asymmetric dimethylarginine (ADMA) were quantified in lung tissue homogenate supernatant using liquid chromatography-mass spectrometry (LC-MS/MS) similarly to previously reported [36]. The ability of NOS to produce NO depends on the availability of substrate l-arginine as well as the competitive inhibitor ADMA. Therefore, the ratio of l-arginine over ADMA (l-arginine/ADMA) was used as a marker of NOS impairment [36,37]. The ratio of l-ornithine over l-citrulline (l-ornithine/l-citrulline) can be used as a marker of the balance between l-arginine metabolizing enzymes [38], as l-ornithine is the product of arginase and l-citrulline the product of NOS activity.

Real-time polymerase chain reaction (rt-PCR) was used to quantify gene expression for arginase and NOS isoforms, in comparison with the average expression of the hypoxanthine guanine phosphoribosyl transferase (Hprt) gene [39]. All reagents were purchased from Invitrogen (Waltham, MA). Quantitative real-time PCR amplification was done with complementary DNA, primers, SYBR Green PCR Master mix with Ampli Taq Gold Polymerase. Primer sets for arginase and NOS isoforms used for amplifications are shown in Table 1. The quality of RNA was assessed based on the ratio of 28S/18S for the quality of agarose denaturing gel electrophoresis and the ratio of A 260/A 280 for spectrophotometry. All PCR protocols included a 3-min polymerase activation step followed by 45 cycles consisting of a 95 °C denaturation step for 30 s, annealing at 60 °C for 30 s, and an elongation step at 72 °C for 30 s. A negative control without template was included for each PCR analysis. Data were collected and analyzed with the provided application software.

## 3. Statistics

Values are presented as mean (±SEM) unless stated otherwise. Statistical analysis was performed by software package in Prism 5 (GraphPad Software, San Diego, CA, USA). One-way ANOVA was used for comparison between three groups or more. Comparisons between specific groups were performed using Tukey post-test or with Kruskal–Wallis test and Dunn’s post-test based on the distribution of data. *p* values <0.05 in differences were considered significant.

## 4. Results

### 4.1. NOx and l-Citrulline Concentrations in the Lung Are Decreased after EVLP

**Figure 1:**
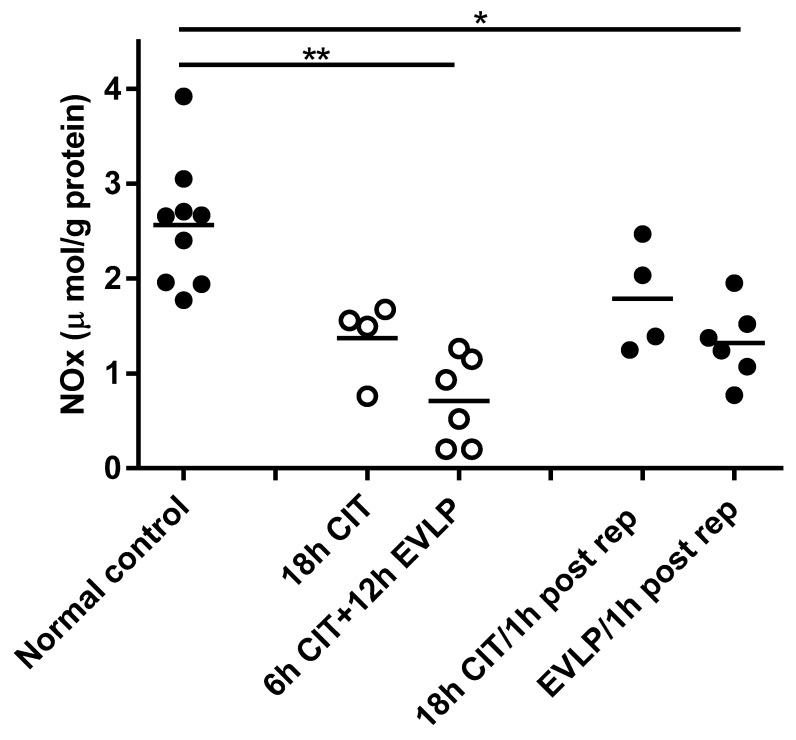
Concentration of nitric oxide metabolites nitrate + nitrite (NOx) in lung tissue homogenates. Each symbol represents one animal lung. Solid symbols indicate samples containing blood, hollow symbols blood-free samples. Lung samples were taken at different time points: normal control, naive recipient left lung; 18 h cold ischemia time (CIT), 18 h cold ischemia time; 6 h CIT+12 h ex vivo lung perfusion (EVLP), 6 h cold ischemia time followed by 12 h EVLP; 18 h CIT/1 h post rep, 1 h after transplantation and reperfusion in the 18 h CIT group; EVLP/1 h post rep, 1 h after transplantation and reperfusion in the EVLP group. ANOVA revealed significant differences between groups (*p* = 0.0006, Kruskal–Wallis test). Post hoc analyses showed significant decreases in the EVLP group before (**, *p* = 0.0004) and after (*, *p* = 0.0016) reperfusion compared to normal control, respectively.

NOx (nitrate + nitrite) tissue concentrations were not different between normal control lungs (2.6 ± 0.2 µmol/g protein) and donor lungs immediately after flush (0 h CIT) (2.1 ± 0.4 µmol/g protein). Lung NOx levels were significantly decreased in the EVLP group at the end of EVLP (6 h CIT+12 h EVLP, 0.7 ± 0.2 µmol/g protein, *p* = 0.0004) and one hour after transplantation (EVLP/1h post rep, 1.3 ± 0.2 µmol/g protein, *p* = 0.0016), compared to normal control. A trend towards lowered NOx concentrations was also seen in the no-EVLP group, which, however, did not reach statistical significance (Figure 1).

**Figure 2:**
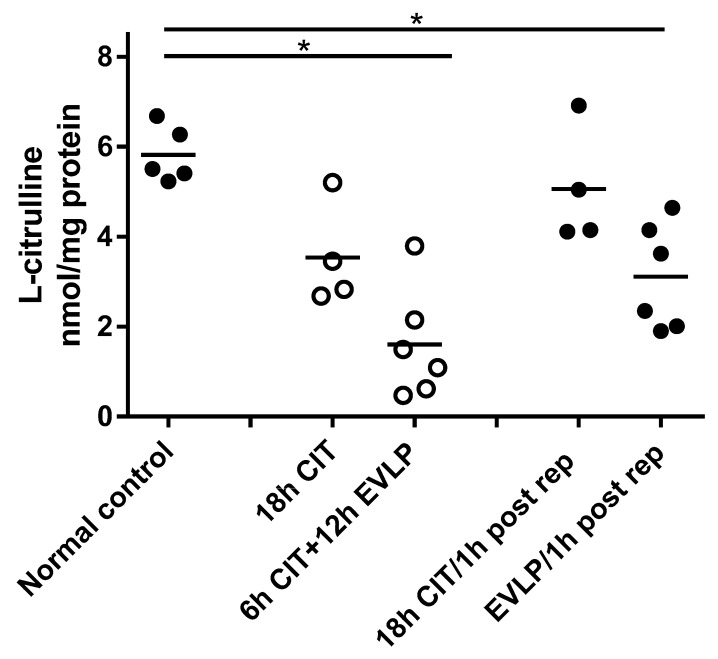
Concentration of l-citrulline in lung tissue homogenates. Each symbol represents one animal lung. Solid symbols indicate samples containing blood, and hollow symbols indicate blood-free samples. Lung samples were taken at different time points: normal control, naive recipient left lung; 18 h CIT, 18 h cold ischemia time; 6 h CIT+12 h EVLP, 6 h cold ischemia time followed by 12 h EVLP; 18 h CIT/1 h post rep, 1 h after transplantation and reperfusion in the 18 h CIT group; EVLP/1 h post rep, 1 h after transplantation and reperfusion in the EVLP group. ANOVA revealed significant differences between groups (*, *p* = 0.0018, Kruskal–Wallis test). Post hoc analyses showed significant decreases in the EVLP group before and after reperfusion (*p* = 0.0043, respectively, Mann–Whitney test), when compared to normal control.

Concentrations of l-citrulline showed similar differences between the groups as seen for NOx. l-citrulline was lowest at the end of EVLP and remained lower than normal after reperfusion. No statistically significant change in l-citrulline was found in the no-EVLP group either after 18 h CIT or one hour after transplantation (Figure 2). As both NO and l-citrulline are products of NOS, these data together suggest that NOS activity was decreased following EVLP.

### 4.2. L-arginine Availability and NOS Impairment

**Figure 3:**
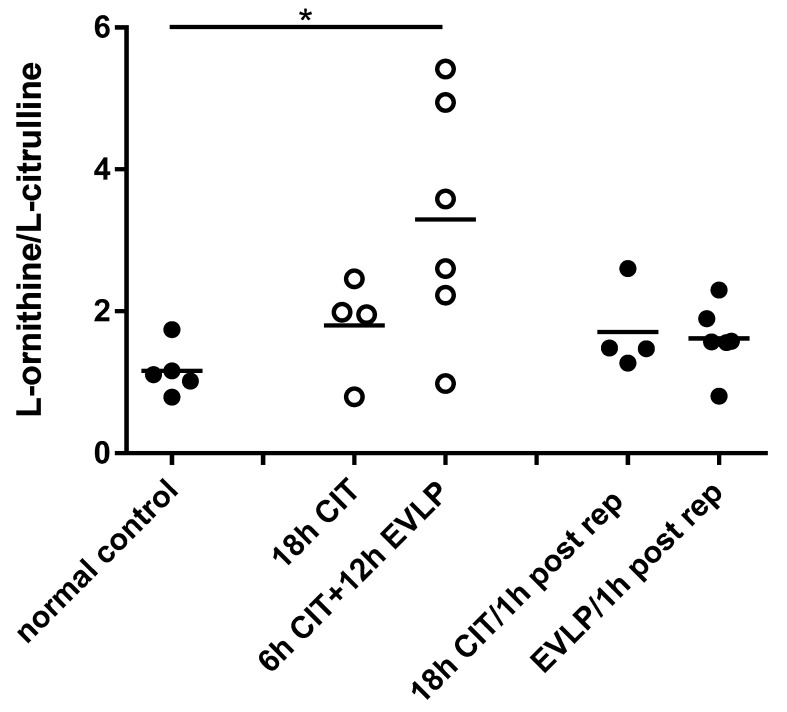
The l-ornithine/l-citrulline in lung tissue homogenates. Each symbol represents one animal lung. Solid symbols indicate samples containing blood, and hollow symbols indicate blood-free samples. Lung samples were taken at different time points: normal control, naive recipient left lung; 18 h CIT, 18 h cold ischemia time; 6 h CIT+12 h EVLP, 6 h cold ischemia time followed by 12 h EVLP; 18 h CIT/1 h post rep, 1 h after transplantation and reperfusion in the 18 h CIT group; EVLP/1h post rep, 1 h after transplantation and reperfusion in the EVLP group. ANOVA revealed a *p*-value suggestive of differences between groups (*p* = 0.078, Kruskal–Wallis test). The ratio was higher in the EVLP group before reperfusion compared to normal control (*, *p* = 0.0226, t-test).

There were no differences in l-arginine (substrate for NOS) or ADMA (NOS inhibitor) concentration nor in the l-arginine/ADMA ratio that would explain the observed decrease in NOS activity (Table 2). In contrast, the l-ornithine/l-citrulline ratio was significantly increased in the EVLP group prior to reperfusion compared to control (3.29 ± 0.69 vs. 1.16 ± 0.16, *p* = 0.0226) (Figure 3). This suggests that EVLP results in a shift of the balance between l-arginine metabolizing NOS and arginase enzymes towards arginase.

### 4.3. NOS and Arginase mRNA Expression

**Figure 4:**
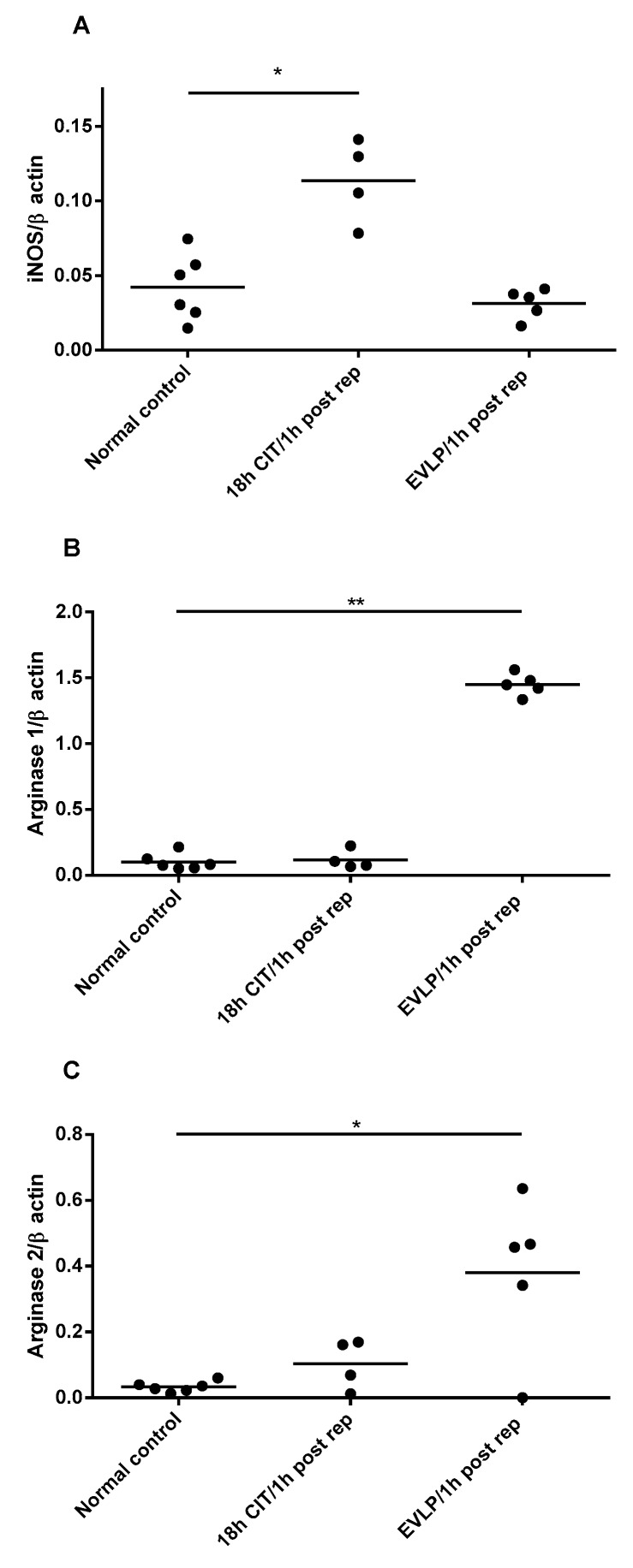
mRNA expression of l-arginine metabolizing enzymes. Expression of **A**) inducible nitric oxide synthase (iNOS), **B**) arginase 1 and **C**) arginase 2 in pig lung. Each symbol represents one animal lung. rtPCR was performed at different time points: normal control, naive recipient left lung; 18 h CIT/1 h post rep, 1 h after transplantation and reperfusion in the 18 h CIT group; EVLP/1 h post rep, 1 h after transplantation and reperfusion in the EVLP group. The expression of iNOS was increased in the 18 h CIT but not the EVLP group. In contrast, the expression of arginase 1 and arginase 2 was increased in EVLP but not the timed controls. (*, *p* < 0.01 and **, *p* < 0.0001, t test compared to normal control, respectively).

Compared to normal control, the expression of iNOS mRNA was increased one hour after reperfusion in the no-EVLP group but not in the EVLP group. In contrast, the expression of both arginase isoforms was significantly increased one hour after reperfusion in the EVLP group but not in the no-EVLP group (Figure 4). No changes in the expression of the nNOS or eNOS were seen (data not shown).

## 5. Discussion

Our data show that NOx levels in lung tissue after 6 h CIT and 12 h EVLP were significantly reduced when compared to normal control lung, and remained decreased after 1 hr of reperfusion. Perfadex flush (0 h CIT) had no effect on NOx tissue levels. The timed controls (18 h CIT) also showed a decrease in NOx levels, which was, however, less pronounced compared to EVLP. Tissue concentrations of l-citrulline followed a similar pattern as seen for NOx, with the lowest levels in the EVLP group before and after 1 h reperfusion. As NO and l-citrulline are both products of l-arginine metabolism from NOS, these data, therefore, suggest that EVLP preservation resulted in reduced NOS activity and reduced NO formation in lung.

Previous studies had reported low NO production after lung transplantation in humans [11,40]. Studies on the effects of preservation and lung transplantation on NO in animal models revealed similar results. For instance, hypothermic preservation for 6 h resulted in reduced NO tissue production in the rat lung [41]. Interestingly, in a study investigating effects of ischemia and reperfusion on NO metabolism in a rat lung injury model for transplantation, while 12 h CIT did not affect NOS expression in the lung, iNOS and eNOS mRNA and protein expression were increased significantly after two hours of reperfusion. Surprisingly, increased NOS expression did not result in an improvement of the lowered tissue NOS activity [40]. This study, therefore, suggested that factors other than changes in NOS expression were responsible for the NO deficiency after lung transplantation. Similarly, in our experiments, 18 h CIT resulted in an increase in lung iNOS expression one hour after reperfusion compared to normal control, but NOx metabolite concentrations were not increased. iNOS mRNA levels after one hour of reperfusion were also significantly higher in the 18 h CIT controls compared to EVLP. Therefore, our results extend previous observations by showing that 6 h CIT followed by EVLP in the pig prevented the increase in NOS expression observed in controls, but also resulted in decreased NO production in the lung.

**Figure 5:**
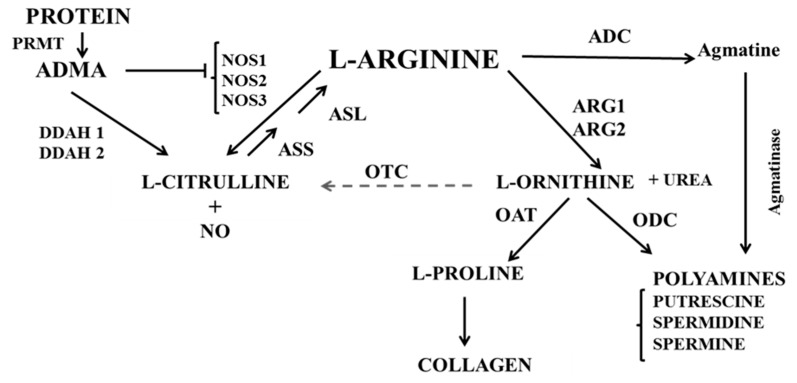
Balance of the l-arginine/NO metabolism between NOS and arginase. ARG, arginase (EC 3.5.3.1); OTC, ornithine carbamoyltransferase (EC 2.1.3.3); ODC, ornithine decarboxylase (EC 4.1.1.17); ADC, arginine decarboxylase (EC 4.1.1.19); NOS, nitric oxide synthase (EC 1.14.13.39); OAT, ornithine aminotransferase 2.6.1.13); OTC, Ornithine transcarbamoylase (EC2.1.3.3); ASS: argininosuccinate synthetase (EC 6.3.4.5), ASL: argininosuccinate Lyase (EC 4.3.2.1), DDAH, dimethylarginine dimethylaminohydrolase; ADMA, asymmetric dimethylarginine; NO, nitric oxide; PRMT, Protein arginine methyltransferases.

There was no evidence from our study that the observed decrease in NOS activity can be explained by reduced concentrations of substrate l-arginine or the endogenous NOS inhibitor ADMA. However, the expression of both arginase isoforms, arginase 1 and arginase 2, was significantly increased after reperfusion in the EVLP group but not in the no-EVLP controls. Arginase is an enzyme of the urea cycles that is also expressed in non-hepatic cells, and among other functions, is known to control NO synthesis by limiting the availability of l-arginine for NOS [37,42,43,44]. Our data, therefore, suggest that the EVLP-induces reduction in NO was secondary to an increase in the expression of arginase. This is supported by the observation that the l-ornithine/l-citrulline ratio was significantly increased after EVLP. The l-ornithine/l-citrulline ratio is an established marker of the balance of the l-arginine metabolism, and increased ratios indicate a shift towards arginase activation [38]. In addition to its regulatory role on NO production, arginase is also thought to contribute to tissue remodelling and fibrosis, as l-ornithine, the product of arginase activity, is a substrate for polyamine biosynthesis and for l-proline production, a precursor of collagen [3,4] (Figure 5). In a rat model of acute allograft rejection after lung transplantation, characterized by increased collagen deposition and high peak airway pressure, increased arginase 1 and 2 protein expression and increased total arginase activity were also found. Interestingly, treatment with the antifibrotic drug pirfenidone, which inhibits arginase activity, prevented the observed tissue remodelling [45]. Recently, in a study of human lungs deemed unsuitable for transplantation, lung function and dynamic compliance was improved by administration of arginase inhibitor, ABH (2(S)-amino-6-boronohexanoic acid), during EVLP [46]. Although physiological conditions are maintained by normothermic EVLP [22,23,24,25,47], EVLP may not prevent alterations in lung tissue cellular metabolism over time [48]. Our study now demonstrates that EVLP induced the expression of arginases, a process that may contribute to the decrease in pulmonary NO production after lung transplantation.

There are a number of limitations in this study. The relevance of the observed increase in arginase expression and decrease in NOS activity following EVLP in pig lung for human transplant recipients remains unclear. However, as previously demonstrated by George et al. in humans [46], arginase may represent a therapeutic target to prevent NO deficiency early after transplantation, and inhibition of arginase, for instance, may result in even better outcomes of lung transplantation after EVLP. In addition, the l-arginine/NO metabolism is complex, and other factors may also have contributed to the observed decrease in NO in the lungs, which were not the focus of our current studies. For example, deficiency of co-factors or changes in pH [49,50,51,52] would also cause NO deficiency. Whether the observed changes in the pulmonary l-arginine/NO metabolism will affect short- or long-term outcomes after transplantation will need to be addressed in future studies. Similarly, whether correction of the observed NO-deficiency by means of therapeutic interventions will result in favourable transplant outcomes was also beyond the focus of our work. Further limitations of this study include relatively small sample sizes of treatment groups as larger numbers would have, for instance, allowed to better distinguish between the effects of prolonged CIT (18 h) vs. 6 h CIT and EVLP.

Nevertheless, these are promising preliminary results suggesting that EVLP leads to an imbalance of the l-arginine metabolism favouring l-arginine consumption by arginase and causing NO deficiency. Since NO plays an important role in airway and pulmonary vascular resistance, studies are needed to test whether therapeutic interventions aiming to increase NO availability such as NO inhalation, or treatment of the donor lung with NO-donors or arginase inhibitors during and/or after EVLP may affect the EVLP-mediated reduction of pulmonary NO production and related short and long-term functional outcomes.

## Figures and Tables

**Table 1:** PCR Primer Sequences.

	Product Length	Forward Primer 1	Reverse Primer 1
Pig-ARG1: Sus scrofa arginase, liver (ARG1), mRNA	163	ACAATCCATCGGGATCATCGGAGC 24	AGGGACATCAGCAAAGCACAGGT 23
Pig-ARG2: Sus scrofa arginase, type II (ARG2), mRNA	229	TGCATTTGACCCTACCCTGGCT 22	TCCCTCCCTTGTCTGCCCAAAACT 24
Pig –iNOS: Sus scrofa iNOS, mRNA	187	TTTCAGGAAGCATCACCCCCGT 22	TGCATGAGCACAGCGGCAAAGA 22
Pig-eNOS: Sus scrofa nitric oxide synthase 3 (endothelial cell) (NOS3), mRNA	203	TGCGATCCTCACCGCTACAACA 22	TGCTCGTTCTCCAGGTGCTTCA 22
Pig-nNOS: Sus scrofa nitric oxide synthase 1 (neuronal) (NOS1), mRNA	159	ACAAAACTCTGCCCCTCGGTGT 22	ACTTGGACGGGCTGCCATTCTT 22

**Table 2:** Lung tissue concentrations of l-arginine, nitric oxide synthase inhibitor asymmetric dimethylarginine (ADMA) and l-arginine/ADMA ratio.

	N	l-arginine nmol/mg Protein	ADMA nmol/mg Protein	l-arginine/ADMA
Normal control	5	30.8 ± 2.0	0.16 ± 0.01	194.4 ± 20.6
18 h CIT	4	28.4 ± 2.2	0.19 ± 0.04	159.7 ± 26.9
6 h CIT+12 h EVLP	6	42.8 ± 11.6	0.22 ± 0.05	188.1 ± 19.9
18 h CIT/1 h post rep	4	35.2 ± 4.2	0.16 ± 0.03	232.1 ± 26.2
EVLP/1 h post rep	6	32.3 ± 5.2	0.13 ± 0.02	240.5 ± 17.3

Concentrations of l-arginine and ADMA (nmol/mg protein) are shown as mean ± SEM. Normal control, naive recipient left lung; 18 h CIT, 18 h cold ischemia time; 6 h CIT+12 h EVLP, 6 h cold ischemia time followed by 12 h EVLP; 18 h CIT/1 h post rep, 1 h after transplantation and reperfusion in the 18 h CIT group; EVLP/1 h post rep, 1 h after transplantation and reperfusion in the EVLP group. ANOVA revealed no differences for l-arginine, ADMA or the ratio, between groups, respectively.
